# Drug-Associated Acute Kidney Disease: Data From a World Pharmacovigilance Database

**DOI:** 10.7759/cureus.63636

**Published:** 2024-07-01

**Authors:** Alexandre Baptista, Ana Marreiros, Ana Macedo, André Coelho

**Affiliations:** 1 Medicine and Biomedical Sciences, Universidade do Algarve, Faro, PRT; 2 Health and Technology Research Center, Instituto Politécnico de Lisboa, Lisboa, PRT

**Keywords:** pharmacoepidemiology, drug-associated nephrotoxicity, pharmacovigilance, adverse drug reactions, acute kidney disease

## Abstract

Background

Drugs are a frequent cause of nephrotoxicity, especially in the context of acute kidney disease (AKD), with a significant number of cases being drug-associated. The WHO's VigiBase is a powerful tool for identifying drugs described and associated with the development of AKD.

Methods

We retrieved data from the period 1968 to 2022 regarding notifications of adverse drug reactions (ADR). The extracted medications were evaluated for their nephrotoxicity based on the bibliographic score (BS) developed through pre-selected references. The main medications involved were classified as 'non-nephrotoxic', 'potentially nephrotoxic', and 'nephrotoxic'. We utilized the IC_025_ and reporting odds ratio (ROR) disproportionality indexes to study the relationship between medications and the odds of being included in an AKD notification.

Results

During the period, a total of 33,932,051 notifications were obtained, revealing 435,677 cases related to drug-associated AKD following MedDRA term filtering, predominantly affecting males aged 45-64. We identified 8,991 active ingredients or suspected combinations associated with AKD development, with the ATC class A - Alimentary Tract and Metabolism being the most frequently described. Among the medications most strongly associated with this phenotype, classes J and N stood out. Among the most notable medications collected, 8.3% were classified as "non-nephrotoxic," 16.7% as "potentially nephrotoxic," and 75% as "known nephrotoxic." Notable active ingredients included cobicistat + elvitegravir + emtricitabine + tenofovir disoproxil (IC_025_ 8.7; ROR 786.96), inotersen (IC_025_ 7.7; ROR 604.57), emtricitabine + tenofovir disoproxil (IC_025_ 7.9; ROR 432.36), esomeprazole (IC_025_ 6.8; ROR 184.23), and pantoprazole (IC_025_ 6.3; ROR 109.86), with proton pump inhibitors dominating the top four positions among the most frequently involved medications.

Conclusion

AKD is a frequent adverse reaction in VigiBase, with a significantly high reported mortality rate. Evaluation of the notifications revealed medications with a high disproportionality index and a strong association with AKD. We also highlight the potential nephrotoxic role of less suspected medications. This study emphasizes the need to consider AKD as a condition potentially associated with iatrogenic etiology, highlighting various medications and their respective involvement in the various possible manifestations of AKD.

## Introduction

Drug-induced nephrotoxicity is a common cause of renal injury, which can manifest through various distinct phenotypes [1]. The exacerbation of renal function associated with medication use can occur over varying periods of time. This acute worsening can be termed acute kidney disease (AKD) if it occurs within three months of the insult. AKD encompasses any functional or structural conditions with an acute exacerbation of the underlying kidney disease, contrasting with chronic kidney disease (CKD), where such changes persist for more than three months [2]. It is a frequent condition, with estimates suggesting that one in every three episodes is drug-induced, showing a higher prevalence in patients admitted to intensive care units (ICU). Indeed, drug-associated AKD has been described in 14-37% of adults, with figures reaching up to 50% in ICUs [3].

Several medications have been implicated in the exacerbation of renal function, with those most frequently involved being aminoglycosides, non-steroidal anti-inflammatory drugs (NSAIDs), contrast agents, and angiotensin-converting enzyme inhibitors (ACEIs) [4]. However, population-based studies using pharmacovigilance have allowed for the identification of new nephrotoxic medications, as well as a review of the main drugs associated with exacerbating renal function [5]. Pharmacovigilance is an essential tool for ensuring the safety and efficacy of medicines on the market, allowing continuous monitoring that contributes to public health protection and enhances healthcare delivery [6]. This monitoring allows for the creation of large-scale databases, the evaluation of which can enable the identification of associations between medications and specific adverse drug reactions, potentially even suggesting medications that may be considered new nephrotoxins [7]. VigiBase is a database of adverse drug reactions managed by the Uppsala Monitoring Center, a collaborating center of the World Health Organization (WHO), which collects anonymized spontaneous notifications of adverse drug reactions [8].

With this in mind, our primary objective was to systematically review notifications submitted to VigiBase over 54 years, identify the principal medications associated with AKD development, and secondarily utilize disproportionality tools to establish robust associative links. It was also our objective, through a bibliographic score developed by us, to try to identify new nephrotoxic agents associated with the development of AKD. Identifying medications commonly implicated in AKD notifications can enhance prescription practices. Knowledge of drugs frequently associated with drug-induced AKD enables physicians to intensively monitor renal function in susceptible patients, thereby significantly improving patient safety.

## Materials and methods

Our study involved a detailed analysis of the largest global database of ADR notifications, which was conducted with approval from the review board. Data was collected from VigiBase, an extensive database that compiles spontaneous ADR reports from numerous participating countries, ensuring full data anonymity, covering the period from 1968 to 2022. After filtering with the selected MedDRA terms, the notifications were extracted into an xlsx file. We implemented stringent procedures to remove any duplicate notifications and assigned a unique identification number to each report for accurate referencing. This dataset includes the most recent updates and provides comprehensive information for each notification, covering anonymized patient details, notifier information, the severity of the reaction, the implicated drug, and an in-depth description of the reported ADR.

The gathering of notifications was conducted after selecting suitable MedDRA terms. Within these notifications, each drug was identified by its active ingredient, which is in line with the WHODrug nomenclature standards. Furthermore, the drugs were classified into pharmacological categories according to the WHO's Anatomical Therapeutic Chemical (ATC) classification system. This method enabled a structured analysis of the data with respect to distinct pharmacological classifications.

In our research, disproportionality analysis was conducted using both the information component (IC) and the reporting odds ratio (ROR). The IC compares the observed frequency of a specific adverse reaction for a particular medication with the expected frequency of that reaction in the general population. A positive IC indicates that the adverse reaction is reported more frequently than expected for the medication, suggesting a possible association. The IC automatically adjusts the observed frequency with the expected frequency, correcting random variations and reducing the likelihood of false positives caused by statistical fluctuations, thereby highlighting only statistically significant associations. The IC filters out spurious data, identifying and disregarding associations that occur by chance, thus reducing the number of false positives. The IC025, the lower limit of the 95% confidence interval of the IC, provides a measure of certainty that an observed association between a medication and an adverse reaction is not occurring by chance. The IC can be complemented with other tools to enhance data robustness and minimize false positive detection. In this regard, we turned to ROR for medications with IC025>0. The ROR is calculated using an odds ratio to assess the association between a medication and an adverse event, where an ROR>1 suggests a positive association, indicating that the medication-induced adverse reaction occurs more frequently than expected. Therefore, while the IC uses a Bayesian approach to adjust observed and expected frequencies, ROR provides a simple measure of association based on odds.

For the main medications evaluated using IC025 and ROR, a bibliographic score was developed to quantitatively determine the degree of nephrotoxicity evidenced in the literature. Each medication was assessed on a scale from 0 (absence of nephrotoxicity) to 5 (certain nephrotoxicity), with this score representing the sum of evidence found in five predetermined bibliographic sources, namely two databases [9,10], one website [11], and two reference books [12,13]. Subsequently, medications were categorized into three distinct classes: non-nephrotoxic with a bibliographic score (BS) of 0, potentially nephrotoxic with a BS of 1 to 2, and fully recognized as nephrotoxic with a BS of 3 or higher.

## Results

Between 1968 and 2022, VigiBase contained a total of 33,932,051 ADR notifications, from which 435,677 notifications remained after filtering for selected MedDRA terms, involving reports of 8991 different active ingredients or combinations thereof. These data comprised 1.284% of all notifications reported in VigiBase during that period.

The peak of notifications was observed in 2019, constituting 10.9% of all collected reports, predominantly involving male patients with 215,846 (49.5%) notifications and predominantly affecting the age group of 45-64 years, which accounted for 111,348 (25.5%) notifications. This demographic distribution closely aligns with the age brackets observed across the entire VigiBase, where the 45-64 year age group similarly exhibits the highest incidence. The analysis revealed that the majority of the identified medications (75%) are already recognized as nephrotoxins. An additional 16.7% of the medications are considered potential nephrotoxins, while the remaining 8.3% represent drugs with newly identified nephrotoxic potential. The most frequently reported principal and co-reported MedDRA terms are detailed in Table 1. Among the pharmacological classes, Anatomical Therapeutics Classification (ATC) class A - Alimentary Tract and Metabolism - was predominant, with 113,472 (26%) of the notifications (Table 2). Notably, omeprazole was the principal active ingredient reported, representing 29,919 (6.9%) of the notifications (see Table 3). Indeed, the top four medications most frequently reported in association with AKD belonged to the proton pump inhibitors class, with omeprazole leading (n=3805), followed by esomeprazole (n=3742), lansoprazole (n=3617), and pantoprazole (n=3277).

**Table 1:** Most reported main and concomitant MedDRA terms in spontaneous notifications of drug-associated acute kidney disease

Main reported MedDRA terms	Number of notifications	Percentage of notifications	Concomitantly reported MedDRA terms	Number of notifications	Percentage of notifications
Acute kidney injury	155,606	35.7	Chronic kidney disease	41012	9.4
Renal failure	110,587	25.4	Diarrhea	19669	4.5
Renal impairment	79,817	18.3	Hypotension	18234	4.2
Blood creatinine increased	64,097	14.7	Nausea	18064	4.1
Renal injury	26,355	6.0	Vomiting	17414	4.0
Blood urea increased	21,556	4.9	Dehydration	16455	3.8
Tubulointerstitial nephritis	16,832	3.9	Dyspnoea	15109	3.5
Glomerular filtration rate decreased	9049	2.1	Pyrexia	15062	3.5
Oliguria	8292	1.9	Fatigue	14984	3.4
Anuria	7047	1.6	Pain	14890	3.4

**Table 2:** Most reported ATC classes (WHO drug classification) in spontaneous notifications of drug-associated acute kidney disease ATC - Anatomical Therapeutics Classification

Anatomical therapeutics classification	Number of notifications	Percentage of notifications
ATC: A Alimentary tract and metabolism	113,472	26.0
ATC: L Antineoplastic and immunomodulating agents	110,485	25.3
ATC: J Antiinfectives for systemic use	110,376	25.3
ATC: C Cardiovascular system	89,394	20.5
ATC: S Sensory organs	73,711	16.9
ATC: D Dermatologicals	55,891	12.8
ATC: M Musculo-skeletal system	45,121	10.4
ATC: N Nervous system	45,118	9.8
ATC: B Blood and blood-forming organs	36,904	8.5
ATC: G Genito urinary system and sex hormones	28,532	6.5
ATC: R Respiratory system	25,560	5.9
ATC: V Various	19,282	4.2
ATC: H Systemic hormonal preparations, excl. sex hormones and insulins	14,632	3.3
ATC: P Antiparasitic products, insecticides and repellents	2862	0.7

**Table 3:** Most reported active ingredients (WHO Drug classification) in spontaneous notifications of drug-associated acute kidney disease (top 10)

Reported active ingredients (WHODrug)	Number of notifications	Percentage of notifications
Omeprazole	29,919	6.9
Esomeprazole	28,671	6.6
Lansoprazole	26,163	6.0
Pantoprazole	23,174	5.3
Emtricitabine + tenofovir disoproxil	15,541	3.6
Tenofovir disoproxil	14,128	3.2
Dexlansoprazole	12,733	2.9
Vancomycin	10,808	2.5
COVID-19 vaccine	10,763	2.5
Furosemide	9322	2.1

However, it is crucial to emphasize additional drugs that, although not among the most frequently reported, exhibit a high risk of association with AKD based on the IC_025_ disproportionality index and ROR. Noteworthy examples among those with the highest disproportionality score include the drug combination cobicistat + elvitegravir + emtricitabine + tenofovir disoproxil (IC_025_ of 8.7; ROR 786.96), emtricitabine + tenofovir disoproxil (IC_025_ of 7.9; ROR 432.36), inotersen (IC_025_ of 7.6; ROR 604.57), tenofovir disoproxil (IC_025_ of 6.0; ROR 90.72), colistin (IC_025_ of 5.7; ROR 66.70), and sodium phosphate (IC_025_ of 5.7; ROR 57.80; refer to Table 1). Drugs most frequently reported such as omeprazole (IC_025_ of 4.8; ROR 6.91), esomeprazole (IC_025_ of 6.8; ROR 184.23), pantoprazole (IC_025_ of 6.3; ROR 109.86), and the combination emtricitabine + tenofovir disoproxil (IC_025_ of 7.9; ROR 432.36) also displayed high IC_025 _indices, indicating a substantial association with AKD (Table 4).

**Table 4:** Main drugs reported by number of notifications, disproportionality indexes (IC025 and ROR), and bibliographic score (BS) AKI - acute kidney injury; BS - bibliographic score; GFR - glomerular filtration rate; PCr - plasma creatinine; TIN - tubulointerstitial nephritis

Active ingredient	ATC Class	Number of reports	Phenotype	IC_025_	ROR	BS
Esomeprazole	A	9198	Renal injury	6.8	184.23	4
Pantoprazole	A	3403	TIN	6.3	109.86	4
Tenofovir disoproxil	J	6461	Renal failure	6.0	90.72	4
Efavirenz + emtricitabine + tenofovir disoproxil	J	4359	Renal failure	5.9	76.08	4
Colistin	J	956	PCr increased	5.7	66.70	4
Furosemide	C	5935	AKI	3.9	17.72	4
Tacrolimus	L	2115	PCr increased	3.6	13.12	4
Omeprazole	A	456	TIN	6.0	6.91	4
Ibuprofen	M	625	TIN	2.6	6.76	4
Emtricitabine + tenofovir disoproxil	J	7781	Renal injury	7.9	432.36	3
Sodium Phosphate	A	728	AKI	5.4	57.8	3
Basiliximab	L	85	TIN	4.7	41.53	3
Spironolactone	C	2644	AKI	4.3	22.28	3
Vancomycin	J	3395	PCr increased	5.8	20.73	3
Canagliflozin	A	1801	AKI	4.0	18.48	3
Acyclovir	J	2097	AKI	3.6	13.68	3
Lenalidomide	L	3507	Renal failure	1.7	3.5	3
Methotrexate	L	2654	AKI	1.5	2.93	3
Inotersen	N	182	GFR decreased	7.7	604.57	2
Ciclosporine	L	1495	PCr increased	3.4	11.86	2
Rivaroxaban	L	3425	AKI	2.1	4.64	2
Cobicistat + elvitegravir + emtricitabine + tenofovir disoproxil	J	3105	Renal Injury	8.7	786.96	1
Metformine	A	5232	AKI	3.5	9.92	0
COVID-19 vaccine	J	5407	AKI	-2.0	0.24	0

Regarding the outcomes, 336,140 (77.1%) of the notifications were classified as 'serious,' with this classification primarily based on the development of 'other medically important conditions' with 192,267 (44.1%) notifications, as detailed in Table 5. The 'fatal' outcome occurred in 59,680 (13.7%) notifications, predominantly affecting male patients (51.2% of notifications), especially those over 75 years old (23.2%). The ATC class L - Antineoplastic and Immunomodulating Agents - was the most reported in notifications with a fatal outcome, accounting for 19,454 (32.7%) of such cases (Table 6). Individually, drugs such as omeprazole, esomeprazole, lansoprazole, and pantoprazole continued to be the most frequently reported suspects in fatal cases, accounting for 3805 (6.4%), 3742 (6.3%), 3617 (6.1%), and 3277 (5.5%) of the notifications respectively (Table 7).

**Table 5:** Severity criteria used for classifying reported notifications

Seriousness criteria	Number of notifications	Percentage of notifications
Other medically important condition	192,267	44.1
Caused/prolonged hospitalization	176,754	40.5
Death	50,019	11.5
Life-threatening	28,696	6.6
Disabling/incapacitating	11,222	2.6
Congenital anomaly/birth defect	533	0.1

**Table 6:** Most reported ATC classes (WHO Drug classification) in fatal spontaneous notifications of drug-associated acute kidney disease ATC - Anatomical Therapeutics Classification

Drug (WHO Drug)	Number of notifications	Percentage of notifications
ATC: L Antineoplastic and Immunomodulating Agents	19,454	32.7
ATC: A Alimentary Tract and Metabolism	14,051	23.6
ATC: C Cardiovascular System	10,895	18.3
ATC: J Antiinfectives for Systemic Use	10,266	17.3
ATC: B Blood and Blood Forming Organs	9206	15.3
ATC: S Sensory Organs	8861	14.9
ATC: D Dermatologicals	7521	12.6
ATC: N Nervous System	7298	12.3
ATC: M Musculo-Skeletal System	6028	10.1
ATC: R Respiratory System	3843	6.5
ATC: G Genito Urinary System and Sex Hormones	3477	5.8
ATC: H Systemic Hormonal Preparations, excl. Sex Hormones and Insulins	3237	5.4
ATC: V Various	2720	4.6
ATC: P Antiparasitic Products, Insecticides and Repellents	453	0.8

**Table 7:** Most reported active ingredients (WHO Drug classification) in fatal spontaneous notifications of drug-associated acute kidney disease (top-10)

Active ingredient	Number of notifications	Percentage of notifications
Omeprazole	3805	6.4
Esomeprazole	3742	6.3
Lansoprazole	3617	6.1
Pantoprazole	3277	5.5
COVID-19 vaccine	2331	3.9
Dexlansoprazole	1863	3.1
Lenalidomide	1715	2.9
Aprotinin	1715	2.9
Rivaroxaban	1391	2.3
Paracetamol	1280	2.2

## Discussion

This analysis of the WHO pharmacovigilance database suggests an association between several medications and AKD based on notifications collected over a period of 54 years.

The results highlight the common occurrence, higher reporting, or lower underreporting rates of drug-associated AKD in VigiBase, accounting for nearly 1.3% of all notifications over the collected period. These data also underscore the serious outcome of these conditions, where 77.1% were considered serious notifications, with a notable mortality rate associated with drug-associated AKD, reported in 13.7% of notifications.

For this analysis, we conducted a descriptive analysis and utilized two disproportionality indices to confirm results, using ROR as a confirmation for IC025 values.

In addition to using these disproportionality indices for the main reported medications, we evaluated these medications using a bibliographic score to assess the degree of nephrotoxicity among them.

Most of the medications identified had their nephrotoxicity supported by clinical studies, case reports, or cohorts, along with reports from institutions such as the European Medicines Agency (EMA). Indeed, the majority of the evaluated medications had a BS greater than three, indicating that they are already well-established nephrotoxic drugs.

Among these, with the highest ROR and IC025 values, are proton pump inhibitors (PPIs), specifically esomeprazole and pantoprazole, which had IC025 values of 6.8 and 6.3, and ROR values of 184.23 and 109.86, respectively. Proton pump inhibitors (PPIs) are indeed one of the most commonly prescribed drug classes worldwide [14]. They have numerous references confirming their well-established nephrotoxic role, particularly tubulointerstitial nephritis, which more commonly presents as acute kidney disease [15].

Several combinations of antivirals also showed non-significant disproportionality indices. Among these, we highlight efavirenz + emtricitabine + tenofovir disoproxil and emtricitabine + tenofovir disoproxil with IC025 of 5.9 and 7.9, and ROR of 76.08 and 432.36, respectively. The former, Atipla®, is a known nephrotoxin with acute kidney injury developing due to drug overload, similar to the latter, commercially known as Truvada®, which is a well-established nephrotoxin, used in both pre-exposure prophylaxis and HIV infection treatment. These combinations are associated with various adverse effects, including rare instances of renal toxicity, primarily attributed to tenofovir [16]. A Brazilian study aimed at evaluating glomerular filtration rate (GFR) evolution in patients over 18 years old receiving daily pre-exposure prophylaxis (PrEP) administration revealed that, as early as the fourth week of administration, over 23% of participants experienced a reduction in GFR exceeding 10 ml/min/1.73 m2 [17]. 

Among the various well-known nephrotoxic medications (BS≥3) with the highest disproportionality indices found, notable examples include colistin (IC025 5.7, ROR 66.70), basiliximab (IC025 4.7, ROR 41.53), spironolactone (IC0254.3, ROR 22.28), vancomycin (IC025 5.8, ROR 20.73), canagliflozin (IC025 4.0, ROR 18.48), acyclovir (IC025 3.6, ROR 13.68), among others, highlighting the predominant role of ATC classes L and J in drug-associated AKD. 

However, it is among the medications with lower BS that we find those with higher IC025 and ROR. On one hand, there is cobicistat + elvitegravir + emtricitabine + tenofovir disoproxil (Stribild®), with an IC025 of 8.7 and ROR of 786.96 for the term 'renal injury'. This is another drug used in the treatment of HIV infection that presents similar limitations and toxicities to emtricitabine + tenofovir disoproxil. However, its high IC025 ROR suggests a strong association with AKD, which is corroborated by its BS of 3.

Another identified drug with a high association was Inotersen, with an IC025 of 7.7 and ROR of 604.57, which has adverse reactions that have prompted the FDA to issue a black box warning for known toxicities. Within the scope of nephrotoxicity, glomerulonephritis and renal failure are recognized entities associated with this molecule [18], and it has recently been linked to the development of segmental and focal glomerulosclerosis [19].

In this study, among the main drugs evaluated with BS, only two drugs showed a BS of 0 - metformin and the COVID-19 vaccine. Metformin, a widely used oral anti-diabetic medication, showed an IC025 of 3.5 and ROR of 9.92 for the development of acute kidney injury, demonstrating a moderate association with this reaction. Indeed, metformin is known to be linked to metformin-associated lactic acidosis (MALA), even when used at normal daily doses [20]. Given the known role of metformin in protecting renal tubular cells from inflammation, apoptosis, reactive oxygen species, etc. [21], studies are being developed to evaluate its potential role as a preventive therapy for acute kidney injury associated with sepsis [22]. In this sense, several authors have questioned the direct involvement of metformin in the development of acute kidney injury [23], although its association with lactic acidosis is well recognized.

From the perspective of the COVID-19 vaccine, it has been shown that its association with increased plasma creatinine is much lower compared to other drug adverse reactions, demonstrating an IC025 of -2.0. However, despite our BS being null, reports can be found regarding the assessment of this vaccine's administration with the development of AKI, as seen in the Taiwanese study that evaluated 27 AKI patients with renal biopsy, showing a high Naranjo score, 16 of whom had associated glomerular disease [24]. These findings have been echoed by others with larger populations, such as the study by Luo et al., demonstrating an increased incidence of AKI following the COVID-19 vaccine, primarily Pfizer, and predominantly in elderly patients [25].

Despite all these medications being associated with the development of acute kidney disease, they were reported using different MedDRA terms. Indeed, with the MedDRA term 'acute kidney injury,' the main reported medications were Sodium phosphate (IC025 5.4), pantoprazole (IC025 5.3), esomeprazole (IC025 5.1), hydrochlorothiazide + olmesartan (IC025 5.0), and omeprazole (IC025 4.8). For the MedDRA term 'anuria', the main associated medications were gentamicin (IC025 3.6), noradrenaline (IC025 3.6), metformin (IC025 3.5). For the terms 'glomerular filtration rate decreased' and 'creatinine renal clearance decreased', medications such as Inotersen (IC025 7.5), remdesivir (IC025 4.7), emtricitabine + tenofovir disoproxil (IC025 4.6) were reported for the first, and medications like deferasirox (IC025 5.3), tenofovir disoproxil (IC025 5.0), and dabigatran (IC025 3.5) were reported for the latter. Finally, associated with tubulointerstitial nephritis, in addition to the well-known proton pump inhibitors, mesalazine (IC025 5.0), flucloxacillin (IC025 4.9), and basiliximab (IC025 4.7) were highlighted.

Overall, among the most involved medications and those classified regarding their nephrotoxicity, no apparent relationship was observed between IC025 and BS. AKD is an entity that, in this study, was characterized either by a high average IC025, ROR, and BS. 

A multitude of other drugs have shown a high prevalence in the notifications of drug-associated AKD, with several demonstrating a notable reporting disproportion in association with this condition. An in-depth exploration of these alleged associations with AKD is beyond the scope of this article due to its extensive nature.

This study can be compared to a French study from 2021, which also evaluated the most frequently associated drugs with drug-induced (DI)-AKI. Interestingly, the results were entirely different from those found in this work, with drugs from ATC classes C and J being significantly more involved (over 20% of notifications in both), highlighting that drugs from ATC class A (the dominant class in our data) did not account for even 5% of notifications [26]. 

Research into acute kidney injury (or the more reductive form of acute renal injury) associated with medication use took its first organizational step with Mehta et al.'s study [1], which defined the structured phenotypes to be evaluated in population studies. Some authors are already studying and developing predictive models to forecast acute kidney injury in short time frames, allowing for the identification of high-risk patients and risk factors and the implementation of preventive strategies [27]. Other authors have been advancing in the identification of biomarkers that enable early recognition of this type of injury, culminating in the early cessation of the suspected agent [27], while the development of propensity scores has been another explored approach [28].

The comprehensive and global nature of the collected data reinforces the validity of our findings, as they reflect real-world clinical practices and mitigate biases inherent in localized reporting methods. The database's reliance on the MedDRA and WHO Drug dictionaries ensures standardized data processing, minimizing the potential for author bias in data interpretation. The application of the IC025 disproportionality index facilitated the use of robust analytical tools, reducing the likelihood of computational errors or manipulation biases. Covering over 50 years of notifications from more than 180 countries, this extensive dataset enables the identification of a wide array of reported drugs, thereby enhancing the reliability of the IC025 index.

Despite its insights, this study is not without limitations. Firstly, it is based on clinical practice and spontaneous reporting. Therefore, data reflect the nature of these spontaneous notifications, which are contributed by both healthcare professionals and non-professionals, and they do not conclusively establish a cause-and-effect relationship between drug use and ADR. Secondly, given that the database comprises spontaneous notifications, there is no absolute certainty that the observed AKD conditions are definitively attributable to ADR. The data may be subject to reporting biases, potentially skewing towards less anticipated or more severe reactions. Thirdly, the potential underreporting of ADR and the common occurrence of multiple drugs being used concurrently add layers of complexity to the interpretation of our results.

Despite its limitations, this article provides a detailed analysis of the main medications identified as suspects in spontaneous reports of drug-associated AKD. It also highlights the key drugs most strongly linked to the development of this phenotype through the evaluation of disproportionality scores (IC025 and ROR) while suggesting potential new nephrotoxic medications. Knowledge of these drugs and their association with AKD enables clinicians to make more informed prescribing decisions and understand the nephrotoxicity potential of the medications they prescribe, thereby significantly enhancing patient safety.

## Conclusions

This study, which evaluated adverse drug reaction notifications gathered in VigiBase over 54 years, demonstrates not only the frequent occurrence of acute kidney disease (AKD) as an adverse reaction during this period but also its association with high mortality rates. This study enabled the identification of the medications most frequently associated with AKD, as well as those exhibiting higher disproportionality (via IC025 and ROR), utilizing one of the world’s largest databases of adverse drug reactions. These investigations facilitate the recognition of nephrotoxins and enhance understanding of the severity and outcomes of drug-associated reactions. This enables clinicians to prescribe with greater knowledge, thereby improving patient safety and reducing the incidence of AKD associated with medication use. It is crucial for all clinicians to recognize the importance of reporting ADRs and to actively engage in their notification.
